# Feasibility of telephone and computerized cognitive testing as a secondary outcome in an acute stroke clinical trial: A mixed methods sub-study of the AcT Trial

**DOI:** 10.1177/23969873251323171

**Published:** 2025-03-12

**Authors:** Sajeevan Sujanthan, Pugaliya Puveendrakumaran, Katie N Dainty, Morgan Barense, Krista L Lanctot, Adrian M Owen, Nishita Singh, Brian H Buck, Houman Khosravani, Shelagh B Coutts, Mohammed Almekhlafi, Ramana Appireddy, Aleksander Tkach, Jennifer Mandzia, Heather Williams, Thalia S Field, Alejandro Manosalva, Muzaffar Siddiqui, Gary Hunter, MacKenzie Horn, Fouzi Bala, Michael D Hill, Michel Shamy, Aravind Ganesh, Tolulope Sajobi, Bijoy K Menon, Richard H Swartz

**Affiliations:** 1 Faculty of Graduate Studies, Institute of Medical Sciences, University of Toronto, Toronto, ON, Canada; 2 Department of Medicine (Neurology), Hurvitz Brain Sciences Program, SB Centre for Brain Resilience and Recovery, Sunnybrook HSC, ON, Canada; 3 Institute of Health Policy, Management and Evaluation, University of Toronto, Toronto, ON, Canada; 4 Department of Psychology, University of Toronto, Toronto, ON, Canada; 5 Baycrest Health Sciences, Rotman Research Institute, ON, Canada; 6 Department of Psychiatry, and Pharmacology and Toxicology, University of Toronto, Toronto, ON, Canada; 7 Department of Physiology & Pharmacology, Psychology, University of Western, Toronto, ON, Canada; 8 Division of Neurology, Rady Faculty of Medicine, University of Manitoba, Calgary, MB, Canada; 9 Department of Medicine, Division of Neurology, University of Alberta, Edmonton, AB, Canada; 10 Faculty of Medicine, Department of Medicine, Division of Neurology, University of Toronto, Toronto, ON, Canada; 11 Department of Clinical Neurosciences and Hotchkiss Brain Institute, Cumming School of Medicine, University of Calgary, Calgary, AB, Canada; 12 Department of Medicine, Division of Neurology, Queen’s University, Kingston, ON, Canada; 13 Kelowna General Hospital, Kelowna, BC, Canada; 14 London Health Sciences Centre and Western University, London, ON, Canada; 15 Queen Elizabeth Hospital, Charlottetown, PE, Canada; 16 Division of Neurology, University of British Columbia, Vancouver, BC, Canada; 17 Medicine Hat Regional Hospital, Medicine Hat, AB, Canada; 18 Grey Nuns Community Hospital, Edmonton, AB, Canada; 19 University of Saskatchewan, Saskatoon, SK, Canada; 20 Diagnostic and Interventional Neuroradiology Department, University Hospital of Tours, Tours, France; 21 Department of Medicine, University of Ottawa, Ottawa, ON, Canada

**Keywords:** Stroke, clinical trials, cognitive endpoint, telephone assessment, computerized assessment

## Abstract

**Introduction:**

Post-stroke cognitive impairment is associated with impaired quality of life. Remote testing provides a potential avenue to measure cognitive outcomes efficiently.

**Patients and Methods:**

Prospective cognitive outcomes were collected at 90–180 days using both telephone MoCA (T-MoCA; range 0–22; <17 impairment) and Creyos, a computerized cognitive battery. Key variables associated with completion were assessed using logistic regressions. Mixed methods brief structured interviews and exit survey were performed to explore barriers to completing computer testing.

**Results:**

Of 791 potentially eligible patients (mean age 70 ± 14 years), there was low feasibility of remote cognitive testing, with only 401 (51%) completing the T-MoCA, and 242 (31%) completing Creyos. Our regression models show that age (OR_T-MoCA_: 0.95 (95% Confidence Interval (CI): 0.94–0.97); OR_Creyos_: 0.95 (95% CI: 0.94–0.96)), functional impairment (mRS 2–5; OR_T-MoCA_: 0.55 (95% CI: 0.37–0.81); OR_Creyos_: 0.66 (95% CI: 0.44–0.98)), quality of life (EQ-VAS; OR_T-MoCA_: 1.02 (95% CI: 1.01–1.03); OR_Creyos_: OR:1.02 (95% CI: 1.01–1.03)) and length of hospital stay (OR_T-MoCA_: 0.98 (95% CI: 0.96–0.99); OR_Creyos_: 0.97 (95% CI: 0.94–0.99)) predicted both telephone and computer cognitive test completion; computer literacy predicted computer test completion (OR_Creyos_: 1.12 (95% CI: 1.04–1.21)). In interviews, a preference for accessibility of computerized testing was reported.

**Discussion:**

Remote cognitive testing has limited feasibility as a secondary outcome in large acute stroke trials. Patients who are older, with worse quality of life, or severe functional impairment post-stroke are less likely to complete remote cognitive outcomes.

**Conclusion:**

Innovative approaches to post-stroke cognitive outcomes in acute stroke trials are needed.

**Data Access:**

Data available upon request.

## Graphical abstract

**Graphical abstract:**
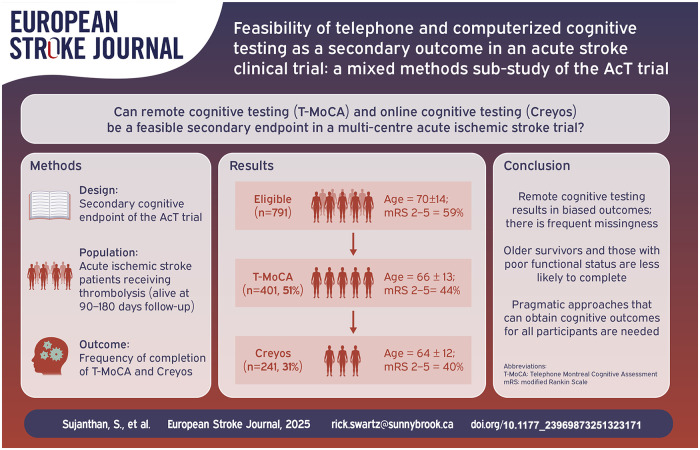

## Introduction

Cognitive deficits are common after stroke, with up to 80% of patients having some degree of cognitive impairment (CI).^1^ Cognitive impairment is often underdiagnosed and under-treated.^2^ Patients with poorer cognition have greater functional impairment and are at a higher risk for both mortality and stroke recurrence.^3^ Despite cognitive impairment being present in many stroke survivors, the primary end points of most randomized controlled trials (RCT’s) in stroke focus on global measures of physical function and rehabilitation,^4^ which do not reflect cognition well.^5^

Though many guidelines have recommended the use of cognitive assessments after stroke both clinically and in stroke clinical trials,^6–8^ they remain infrequently used in trials, with substantial heterogeneity in approaches.^9,10^ Even when embedded in clinical trials, in-person cognitive testing has significant limitations. The Montreal Cognitive Assessment (MoCA)^11^ is a commonly used general cognitive screening tool that assesses multiple domains and has broader application in stroke^12^ than the Mini-Mental State Examination (MMSE).^13^ However, general screens like the MoCA and MMSE exhibit floor- and ceiling-effects, with many stroke survivors in the “intermediate” range, making it difficult to define reliable cut-offs for impairment.^14^ Additionally, these domain-general screens may not be suitable for stroke patients impacted by focal neurological impairments such as aphasia, visual loss, neglect, apraxia, alexia or agraphia, further contributing to a selection bias and attrition.^15^

There is a shift to the use of domain-specific cognitive screens such as the Oxford Cognitive Screen (OCS) which were designed to be more inclusive of stroke patients^15,16^ and to account for stroke focal neurological deficits with aphasia- and neglect-friendly measures.^6^ However, even these assessments are limited by significant attrition, with roughly 50% attrition at 6-month follow-up.^16^

Unlike these cognitive screens, detailed in-person cognitive batteries^17^ can distil key components of full neuropsychological assessments into feasible assessments that capture multiple cognitive domains. While they provide more detailed cognitive characterization, these in-person 30–90-min batteries are not feasible for multi-center international clinical trials. They must be completed by a trained administrator and are time-consuming and costly.^18^

In-person testing of any sort exclude participants with difficulty attending clinics due to physical and cognitive limitations.^18^ This may lead to sampling biases, as those with greater cognitive impairment may be less able or less likely to return for in person assessments or to consent to detailed cognitive testing. Indeed, prior cognitive sub-studies of stroke trials (VITATOPS,^19^ ESCAPE,^20^ SECRET^21^) with in-person cognitive assessments have shown frequent non-random missingness.

The use of cognitive assessments delivered remotely, by telephone or online, has potential to address some of the limitations presented by in-person assessments.^22^ Remote assessments increase convenience for participants, and avoid concerns of travel. Especially since the COVID pandemic, the potential for remote testing has received much more attention.^23^ On an individual participant level, both telephone and online assessments show excellent correspondence to in-person pen-and-paper tasks,^6^ but the feasibility of implementing remote cognitive testing in the context of multi-center stroke clinical trials has not been previously characterized.

## Objective

The objectives of this study were: (1) to investigate the feasibility of remote cognitive testing as a secondary endpoint in a multi-center randomized controlled trial in acute ischemic stroke and (2) to determine quantitative and qualitative factors that relate to remote cognitive test completion.

## Methods

### Patients

All patients were recruited from the Alteplase compared to Tenecteplase (AcT) clinical trial.^24^ AcT randomized all participants with an acute ischemic stroke who met criteria for intravenous thrombolysis^25^ from December 10, 2019 to January 25, 2022. The substudy was initiated after the main trial had begun, and not all sites were involved. To reduce sampling bias, all potential English-speaking consenting participants enrolled in the parent trial (treated with either intravenous alteplase or tenecteplase), who completed the parent trial’s primary outcome independently, and were within 90–180 days from randomization were eligible for this substudy. A single trained assessor (SS) contacted all potentially eligible participants. To minimize attrition, voicemail message were left at the primary contact number (used for main study primary outcome assessment), or alternative contacts where available. After maximum of five voicemail messages without response within the 180-day window, participants were considered lost to follow-up. Participants who could not complete cognitive testing, including those with severe aphasia (unable to talk on the phone) and those who were not fluent in English, were excluded. The substudy was approved by the research ethics board at all sites from which participants were recruited.

### Cognitive assessment

Consenting participants completed the T-MoCA,^26^ a modified version of the MoCA designed for remote/telephone administration.^6^ SS obtained certification from the official MoCA website (mocacognition.com) before assessing participants. After completing the T-MoCA, those able and willing completed an abbreviated Creyos battery.^18,27^ We selected Creyos battery as a standardized, easy to access set of test that have a very large normative dataset,^27–29^ and for which the subtest have been validated against pen-and-paper cognitive tasks.^30–32^ The scores can be combined to form three key factor-based domain scores of interest – Reasoning Ability, Verbal Ability, and Short Term Memory.^27^ Consenting participants received an individualized URL link via email to complete the online assessment. Over the phone, SS ensured the participant could access the assessment, understand how the tests worked and navigate the video instructions and online practice rounds before the participant completed the task independently. If the participants encountered any difficulties, SS was available by phone to provide prompt assistance. Participants without access to a computer or a stable internet connection (home, library, clinic) were not eligible for online testing. The cognitive tasks used for this study included: Paired Associates, Monkey Ladder, Rotations, Odd One Out, Digit Span, and Grammatical Reasoning (creyos.com).

### Structured qualitative exit interviews

Structured qualitative exit interviews^33^ were performed with all consenting participants after attempting the Creyos and were collected from July 2021 to July 2022. The interviews were conducted over the phone and analyzed by trained interviewers (SS & PP). The structured interview guides were designed for specific participant populations: (a) those that successfully completed all Creyos assessments, (b) those that began the online tasks but did not finish all Creyos assessments. The last group (c) who were eligible but did not undertake the Creyos assessments, were only asked a single, multiple select question exit survey (Supplemental Data, Section 2 (Methods – pages 4–5).

### Statistical analysis

Proportion of participants completing telephone and computerize cognitive assessments are reported. Descriptive statistics, including mean and standard deviations, were calculated for continuous variables age, quality of life (EQ-VAS), length of hospital stay, onset to needle time (OTN), door to needle time (DTN), and assessed with t-test (Supplemental Table 1). Proportions were reported for categorical variables such as enrolling center type (primary or comprehensive stroke center), affected hemisphere and occlusion sites, and assessed with a chi-square test (Supplemental Table 1). Logistic regression models were used to examine the association between demographic and clinical variables and completion of remote cognitive testing (yes/no) with independent models for T-MoCA or Creyos completion. Models controlled for age, sex, 90 day follow-up modified Rankin Scale (mRS), treatment type, enrolling center type, registry (QuICR^34^ vs OPTIMIZE,^35^ are Canadian acute stroke registries that collected process of care metrics – QuICR in Alberta and OPTIMIZE in the rest of Canada), baseline National Institutes of Health Stroke Scale (NIHSS), length of hospital stay, process metrics (Onset-to-Needle (OTN)/Door-to-Needle (DTN)), self-rated quality of life (QoL) using EQ-VAS, presence of large vessel occlusion (LVO), affected hemisphere and occlusion site on computer tomography angiography (CTA), ethnicity, education and computer proficiency score.^36^ All models were assessed for multicollinearity using variance inflation factor (VIF) with a cut-off < 5. Only complete observations were utilized in all models, with pairwise deletion. Associations between variables within the models were set at p < 0.05, two-sided test. Statistical analyses were performed using R-programing (version 4.2.3).

### Structured qualitative interview analysis

Structured qualitative interview data were analyzed using a combination of standard thematic analysis technique^37^ to identify themes and content analysis to quantify the frequency of themes related to computerized cognitive assessment. Research team members SS and PP independently reviewed the transcripts, identified sections that reflected the key domains of interest. Subthemes that expressed similar experiential patterns were brought together to develop core themes. The exit survey was analyzed exclusively using content analysis. Additional details are available in the Supplemental Data, Section 2 (Methods – pages 4–5).

## Results

### Completion of cognitive assessments

**Figure 1.**
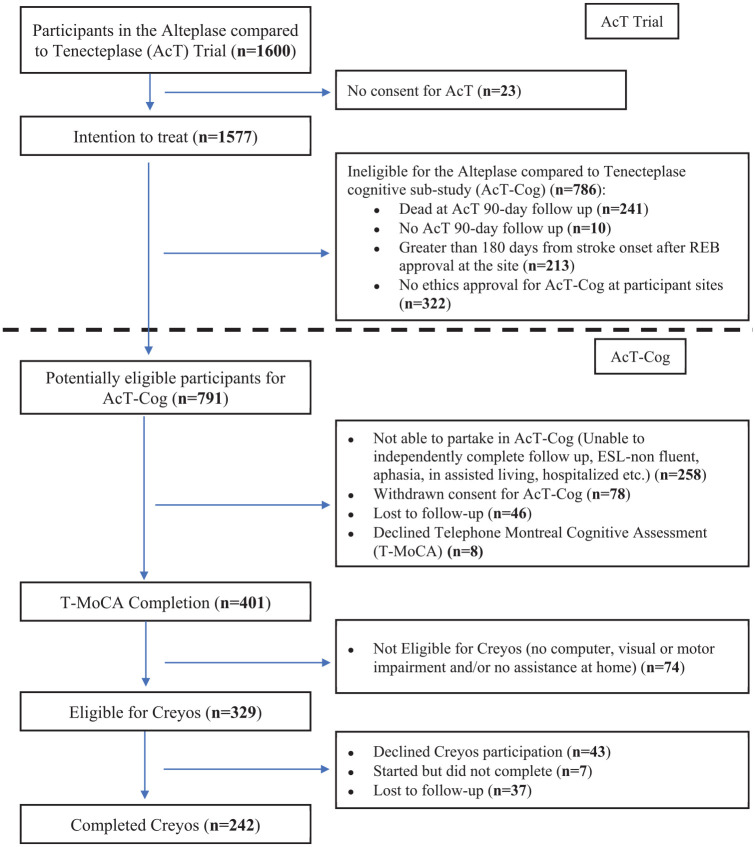
Patient flowchart from the parent Alteplase compared to Tenecteplase (AcT) trial into the cognitive sub-study (AcT-Cog), highlighting the exclusion/missingness.

Amongst the 1577 patients in the intention to treat analysis of the AcT Trial,^24^ 786 (49.8%) were not eligible for the cognitive sub-study (Figure 1). Of the remaining 791 potentially eligible patients for this cognitive sub-study (mean age 70 ± 14 years, and median mRS of 2), 409 (52%) were able to speak independently on the phone and agreed to answer questionnaires. They were all recruited within 180 days from randomization. Of those, 401 participants (51% of the 791 potentially eligible) consented and completed the T-MoCA. Of these 401 participants (mean age 66 ± 13 years), 156 (38.9%) participants were female and 207 (52%) were assigned to tenecteplase. About 242 participants (31% of the 791 potentially eligible; mean age 64 ± 12 years) consented and competed Creyos. Of these, 92 (38%) were female and 124 (51%) were assigned to tenecteplase (Supplemental Table 1). Common reasons for Creyos incompletion included 18.5% of participants needing assistance at home, lacking computer access, or having visual/motor impairments, and 20% of participants declining consent or not returning calls (Figure 1). In univariate analyses, compared to those not completing cognitive testing, participants who completed a T-MoCA or Creyos (Supplemental Table 1) were more likely to be younger, male, with lower baseline stroke severity (NIHSS), have better functional outcome at 90 days (mRS 0–1), have better self-rated QoL at 90-days and have had an earlier discharge from hospital.

### Multivariate analysis of factors associated with T-MoCA and Creyos completion

For all models, occlusion location on CTA was removed as it had a VIF > 5. Table 1 describes the results of the two multivariable logistic regressions exploring the association of T-MoCA and Creyos completion (yes/no) respectively with demographic and clinical variables (n = 791). For T-MoCA, age (OR: 0.95 per age increase, 95% CI: 0.94–0.97), grouped mRS – 2–5 (OR: 0.55 compared to mRS 0–1, 95% CI: 0.37–0.81), EQ-VAS (OR: 1.02 per point increase, 95% CI: 1.01–1.03), and length of hospital stay (OR: 0.98 per 1-day increase, 95% CI: 0.96–0.99) were independently associated with completion. For Creyos, age (OR: 0.95 per age increase, 95% CI: 0.94–0.96), mRS 2–5 (OR: 0.66 compared to mRS 0–1, 95% CI: 0.44–0.98), EQ-VAS (OR: 1.02 per point increase, 95% CI: 1.01–1.03) and length of hospital stay (OR: 0.97 per 1-day increase, 95% CI: 0.94–0.99) were also independently associated with completion.

**Table 1.** Predicting Telephone Montreal Cognitive Assessment (T-MoCA) and Creyos completion in potentially eligible Alteplase compared to Tenecteplase cognitive sub-study (AcT-Cog) participants (n = 791). Determining relationship of T-MoCA and Creyos completion with key demographic and clinical variables.

Term	Odds ratio of T-MoCA completion (95% confidence interval)	Odds ratio of Creyos completion (95% confidence interval)
Sex – Female	0.89 (0.63–1.25)	1.04 (0.73–1.50)
Age	0.95 (0.94–0.97)*	0.95 (0.94–0.96)*
Grouped Modified Rankin Scale – 2–5	0.55 (0.37–0.81)*	0.66 (0.44–0.98)*
Drug - tenecteplase	1.02 (0.73–1.42)	0.95 (0.67–1.35)
Type of Enrolling Center – comprehensive center	0.81 (0.46–1.41)	1.25 (0.70–2.25)
Source – QUICR	0.82 (0.59–1.15)	0.92 (0.65–1.31)
Large vessel occlusion on computed tomography angiography (CTA) – Yes	1.54 (0.93–2.53)	1.48 (0.87–2.51)
Onset-to-needle time (min)	1.00 (1.00–1.00)	1.00 (1.00–1.00)
Door-to-needle time (min)	1.00 (1.00–1.01)	1.00 (1.00–1.01)
EQ – Visual Analog Scale (VAS)	1.02 (1.01–1.03)*	1.02 (1.01–1.03)*
Length of hospital stay	0.98 (0.96–0.99)*	0.97 (0.94–0.99)*
Baseline NIHSS	0.97 (0.94–1.00)	0.97 (0.94–1.00)
Occlusion site on CTA (compared to patients with no visual occlusion on CTA)		
Left	0.90 (0.58–1.39)	0.90 (0.57–1.44)
Right	1.31 (0.80–2.13)	0.98 (0.58–1.64)
Bilateral	2.30 (0.38–13.84)	1.87 (0.36–9.70)
Midline (Brainstem)	1.15 (0.3–4.37)	0.48 (0.12–1.92)

* Significant results (*p* < 0.05) are bolded.

In both models (Table 1) increasing age, more severe functional impairment and longer hospital stays were correlated with lower likelihood of completing the T-MoCA and Creyos, while higher scores on EQ-VAS (better self-rated quality of life) correlated with increased T-MoCA and Creyos completion.

Supplemental Table 2 describes the multivariable logistic regression of the association between demographic and clinical variables and the likelihood of Creyos online cognitive assessment completion (yes/no) in the subset of people who were independently able to speak on the phone (n = 409). In this group, only computer proficiency (OR: 1.12 per point increase, 95% CI: 1.04–1.21) had a significant positive association with completion.

### Structured qualitative interview and exit survey findings

There were two groups of interview participants: (a) those that successfully completed all Creyos assessments (n = 76); (b) those that attempted but did not finish Creyos (n = 3); and the last group (c) those who were eligible but who did not undertake the Creyos assessments, that only completed an exit survey (n = 43; Supplemental Table 3). A code book was developed using a thematic analysis of the Structured Qualitative Interview from groups (a) and (b) (Supplemental Table 4). The qualitative data showed that individuals who were able to complete Creyos preferred online testing due to improved accessibility and comfort. Without an administrator, participants felt they were limited in their opportunities to obtain feedback, had difficulty understanding task objectives and/or had reduced motivation to put forward their best efforts (Supplemental Table 4). Forty-five percent of the interviewed participants reported increased perceived difficulty in tasks (Supplemental Figure 1). Based on the brief exit survey, the most common self-reported reasons for declining participation on computerized testing were computer literacy challenges (49%), along with lack of interest (47%) and time demands of the tasks (44%; Supplemental Figure 2). Additional details regarding the results of the structured qualitative interview and exit survey is available in the Supplemental Data, Section 2 (Results – pages 6–12).

## Discussion

Telephone screening or computerized cognitive assessments are not ideal as a secondary outcome for large acute stroke trials. In clinical trials, the prioritizing of primary outcome data can result in more variability in secondary endpoint data collection, particularly when these require additional participant engagement. In the parent AcT trial, data for the EQ-VAS quality of life at 90-days was missing for n = 74/1577 (5%)^38^; however, this included both surrogate (patient/family) ratings, and imputation of lowest values for those who didn’t survive (n = 241). Thus, even during primary data collection, there can be significant missingness for variables that require direct participant response. For cognitive outcomes, especially requiring a second contact, even more participants declined to participate: whether via telephone (51%) or online (31%), completion rates were moderate and demonstrated survivor biases, with older and more severely affected participants less likely to complete assessments.

Remote cognitive testing has been proposed as an approach to help overcome some of the challenges introduced by in-person screens and gold standard testing including geographic barriers^39^ and economic accessibility.^40^ Our data show that any form of remote cognitive assessment can end up as a barrier to inclusivity and accessibility, with non-random missingness of data: older stroke survivors, those who experience worse quality of life, those with more severe strokes or with poor functional outcomes were less likely to complete our remote cognitive secondary outcomes. Since age and stroke severity are known correlates of cognitive function after stroke,^41^ the people with the greatest cognitive difficulties may also be the least likely to complete cognitive testing. It is likely that inability to complete cognitive testing is, in and of itself, a potential marker of poor post-stroke outcome as reflected by associations with poor functional outcome and quality of life.

The biases seen here do not only apply to hyperacute cohorts, or to remote cognitive testing. Indeed, the completion of remote detailed cognitive assessment reported here (30%) is similar to rates seen in a stable stroke prevention clinic sample^42^ where, out of 1400 patients attending stroke prevention clinics who underwent very brief screening, only 29% agreed to complete a 30 min in-person detailed cognitive assessment. In that sample, those that declined were also older, with greater stroke severity.^42^ Similarly, in the ESCAPE trial,^20^ a 59% completion rate was reported for the in-person MoCA, comparable to our T-MoCA completion rate of 51%, with missing participants tending to be older and having severe stroke impairments. These studies suggest that biases introduced by cognitive testing after stroke is a problem for both acute and prevention trials and for both in-person and remote assessments. In contrast, the secondary prevention LACI-2^43^ trial reported a completion rate of 85% for the T-MoCA. These trials, by nature of their inclusion criteria and prospective design, are by definition restricted to those willing and able to complete cognitive tests at baseline, which improves completion rates; yet, attrition still occurs non-randomly. More broadly, participants with Alzheimer’s disease^44^ or heart failure^45^ who do not complete follow-up cognitive testing are also more likely to be those with poor cognitive and/or functional outcomes. With significant reductions in sample sizes and non-random missingness, cognitive outcomes reported in randomized trials have a high risk of bias, limiting the translational aspect of trial findings.^46^

In stroke clinical trials, measuring cognitive impairment requires direct participant assessment, so is necessarily limited to those who are able and willing to participate.

An alternative approach that identifies meaningful cognitive outcomes for all patients in acute stroke trials is required. One approach, used in the recently published LACI-2^43^ randomized clinical trial, involved a 7-level ordinal cognitive outcome status^47^ designed to reflect DSM-5 criteria for neurocognitive impairment. Each category was operationalized using a combination of T-MoCA and/or the modified Telephone Interview for Cognitive Status (TICS-m) scores,^47^ plus mRS or Barthel functional measures. In this setting, 86% of people completed either a T-MoCA or TICS and a functional outcome and could thus generate an ordinal cognitive outcome. This scale has some limitations – for example, application for individuals with discrepant cognitive and functional status may be inconsistent, and data were still missing in 14% of participants. While the LACI-2 trial utilized validated cognitive screens (T-MoCA and TICS-m) and functional measures (mRS or Barthel), the novel ordinal scale operationalized using various cut-offs has not been validated against gold standard neuropsychological testing. However, it is nonetheless an important attempt to create an ordinal scale that reflects cognitive function and is available in majority of the participants. This represents a necessary progression away from reliance only on direct cognitive screening or standardized cognition as outcomes.

Other, indirect approaches to cognition, such as machine learning analysis of recorded spontaneous speech samples,^48^ in person or video eye-tracking technology,^49^ or a combination of both,^50^ may help to account for participants too impaired for traditional cognitive tasks. Additionally, caregiver or family-reported concerns can provide insights into cognitive problems experienced by the patient.^51^ Indirect measures of cognition from informants include standardized participant or informant rating of cognitive function (e.g. Informant Questionnaire on Cognitive Decline in the Elderly (IQCODE)^52^) and cognitively focused instrumental activities of daily living (e.g. Lawton and Brody Instrumental Activities of Daily Living (IADL)^53^). These validated questionnaires are relatively unaffected by social and cultural biases and can even be compared to a participants baseline to account for pre-stroke function and post-stroke cognitive changes.^54^ Though these assessments do not provide a direct reflection of global cognition or specific cognitive domains, they identify functionally important changes attributable to cognitive impairment.

Cognitive impairments can significantly impact a person’s independence, quality of life and clinical outcomes.^55^ However, there is limited literature on the ideal assessment method and the optimal duration for follow-up within stroke clinical trials.^8^ Our findings indicate that employing remote telephone or computerized cognitive assessment as a secondary endpoint in a large acute clinical trial is heavily influenced by attrition and survivor biases. Future stroke trials aiming to assess cognition should embed cognitive assessments within the primary endpoint visit whenever possible. Additionally, the use of informant-reported questionaries could help to limit missingness and obtain outcomes for all participants – regardless of age and stroke severity.

## Limitations

This study examined a cohort of English-speaking participants enrolled in a time-sensitive acute stroke treatment trial, with cognition as a secondary endpoint. The completion rates reflect the outcomes of hyperacute stroke, with high rates of mortality and functional impairment affecting recruitment and attrition. Further, some potentially eligible patients were missed if they were enrolled in the trial at sites not participating in this substudy, or completed the trial before local ethics approval was obtained for the substudy. Predictor variables were necessarily limited to those available for all participants in the primary trial; hence, some variables, such as primary language spoken or socio-economic status, that were not collected in AcT could not be used in models predicting test completion. Participants had to speak to a coordinator by phone to access the online assessment, potentially limiting participation from those who would prefer to avoid phone calls and access computer testing directly. Additionally, the structured qualitative interviews and exit surveys were also necessarily limited to those who completed the telephone assessment. As with the cognitive testing, the language requirement of the qualitative questions may have limited participant comprehension. It is possible that other telephone or computer assessments may have had different completion rates; however, both the regression data and the qualitative interviews suggest that completion is more related to participant factors than test-specific factors. It is also important to note that while the Creyos online tests are based on validated pen-and-paper tasks, the online assessment themselves have not been directly validated in stroke, and do not cover all domains of stroke-specific cognition. As this substudy required a secondary contact, 1–3 months after the primary study endpoint assessment, it carried a higher risk for attrition.

## Conclusion

To improve cognitive outcomes after stroke, it is essential to include cognitive measures (direct or indirect) as outcome events in clinical trials. Our results reinforce the need for clinical trials to identify more pragmatic cognitive outcome events, that can be collected as a primary or secondary outcome at time of primary assessment, and that can be facilitated or completed by surrogates for more severely affected participants. Further, studies of cognitive outcomes must transparently report rates of missingness, identify reasons for incompletion (declining consent for convenience or lack of time commitment vs lack of capacity) and account for non-random missingness by controlling for key variables identified here, including age, stroke severity, QoL and length of hospital stay.

## Supplemental Material

sj-docx-1-eso-10.1177_23969873251323171 – Supplemental material for Feasibility of telephone and computerized cognitive testing as a secondary outcome in an acute stroke clinical trial: A mixed methods sub-study of the AcT Trial Supplemental material, sj-docx-1-eso-10.1177_23969873251323171 for Feasibility of telephone and computerized cognitive testing as a secondary outcome in an acute stroke clinical trial: A mixed methods sub-study of the AcT Trial by Sajeevan Sujanthan, Pugaliya Puveendrakumaran, Katie N Dainty, Morgan Barense, Krista L Lanctot, Adrian M Owen, Nishita Singh, Brian H Buck, Houman Khosravani, Shelagh B Coutts, Mohammed Almekhlafi, Ramana Appireddy, Aleksander Tkach, Jennifer Mandzia, Heather Williams, Thalia S Field, Alejandro Manosalva, Muzaffar Siddiqui, Gary Hunter, MacKenzie Horn, Fouzi Bala, Michael D Hill, Michel Shamy, Aravind Ganesh, Tolulope Sajobi, Bijoy K Menon and Richard H Swartz in European Stroke Journal
