# Supplementary material for: Feasibility of telephone and computerized cognitive testing as a secondary outcome in an acute stroke clinical trial: A mixed methods sub-study of the AcT Trial
Source: Eur Stroke J. 2025 Mar 12;10(3):968–77. doi: 10.1177/23969873251323171 (PMC11907497; doi:10.1177/23969873251323171)
Supplement: sj-docx-1-eso-10.1177_23969873251323171 – Supplemental material for Feasibility of telephone and computerized cognitive testing as a secondary outcome in an acute stroke clinical trial: A mixed methods sub-study of the AcT Trial [file sj-docx-1-eso-10.1177_23969873251323171.docx]

**Table of Contents**

**Section 1: Supplemental data tables**

1. Supplemental Table 1 - Patient Characteristics 2
2. Supplemental Table 2 - Predicting completion of Creyos online computer battery 3

**Section 2: Structured Qualitative Interview and Exit Survey**

1. Methods:
   1. Qualitative Interview, Exit Survey and Qualitative Analysis Plan 4
   2. Qualitative Interview Questions and Exit Survey Question 5
2. Results:
   1. Brief Structured Qualitative Interview and Exit Survey Results 6
   2. Supplemental Table 3 – Qualitative Interview Patient Demographics 7
   3. Supplemental Table 4 – Codebook developed using a thematic analysis 8
   4. Supplemental Figure 1 – Participants self-reported barriers to performance 12
   5. Supplemental Figure 2 – Participants self-reported reason for declining participation 12
3. References 13

**Section 1: Supplemental data tables**

**Supplemental Table 1**: Patient Characteristics of Telephone Montreal Cognitive Assessment (T-MoCA) and Creyos completed patients (n=791)

|  | **T-MoCA Completion** | |  | **Creyos Completion** | |
| --- | --- | --- | --- | --- | --- |
|  | **No (n=390)** | **Yes (n=401)** |  | **No (n=549)** | **Yes (n=242)** |
| Age (mean (SD)) | **75 (14)** | **66 (13)** |  | **73 (14)** | **64 (12)** |
| Sex (% Female) | **195 (50%)** | **156 (39%)** |  | **259 (47%)** | **92 (38%)** |
| Drug Allocation (% tenecteplase) | 198 (51%) | 207 (52%) |  | 281 (51%) | 124 (51%) |
| Modified Rankin Scale (% 2-5) | **292 (75%)** | **177 (44%)** |  | **372 (68%)** | **97 (40%)** |
| Time from onset-to-needle, mean (SD), mins | 146 (62) | 143 (63) |  | 147 (63) | 140 (62) |
| Time from door-to-needle, mean (SD), mins | 42 (21) | 46 (52) |  | 43 (24) | 47 (62) |
| EQ – Visual Analogue Scale (VAS) (mean (SD)) | **61 (24)** | **74 (19)** |  | **64 (24)** | **76 (17)** |
| Length of Hospital Stay (mean (SD)) | **13 (19)** | **6.7 (7.6)** |  | **12 (17)** | **5.6 (5.6)** |
| Pre-treatment National Institutes of Health Stroke Scale (NIHSS) (mean (SD)) | **11 (7.0)** | **9.5 (6.2)** |  | **11 (6.8)** | **9.2 (6.3)** |
| Type of Stroke center (% Comprehensive center) | 352 (90%) | 352 (88%) |  | 488 (89%) | 216 (89%) |
| Registry (% QUICR) | **244 (63%)** | **220 (55%)** |  | 331 (60%) | 133 (55%) |
| Large Vessel Occlusion on Computed Tomography Angiography (CTA) (% Yes) | 116 (30%) | 126 (32%) |  | 168 (31%) | 74 (31%) |
| Occlusion Side on CTA (%) | | |  |  |  |
| No occlusion seen | 144 (37%) | 154 (39%) |  | 201 (37%) | 97 (40%) |
| Left | 137 (35%) | 115 (29%) |  | 181 (33%) | 71 (30%) |
| Right | 99 (26%) | 115 (29%) |  | 149 (27%) | 65 (27%) |
| Bilateral | 2 (0.5%) | 5 (1.3%) |  | 4 (0.7%) | 3 (1.2%) |
| Midbrain | 5 (1.3%) | 10 (2.5%) |  | 10 (1.8%) | 5 (2.1%) |
| Occlusion Location on CTA (%) | | |  |  |  |
| No occlusion seen | 194 (50%) | 191 (48%) |  | 266 (49%) | 119 (49%) |
| Distal occlusion | 63 (16%) | 73 (18%) |  | 95 (17%) | 41 (17%) |
| M2 Middle Cerebral Artery (MCA) occlusion | 79 (20%) | 72 (18%) |  | 109 (20%) | 42 (17%) |
| M1 MCA occlusion | 54 (14%) | 65 (16%) |  | 79 (14%) | 40 (17%) |

***Significant differences (p < 0.05) are bolded**

**Supplemental Table 2 -** Predicting completion of Creyos online computer battery in Alteplase compared to Tenecteplase cognitive sub-study (AcT-Cog) participants able to speak on the phone (n=409). Determining relationship of Creyos completion with key demographic and clinical variables.

| **Baseline Characteristics** | **Odds Ratio (95% CI)** |
| --- | --- |
|  |  |
| Sex – Female | 0.97 (0.45 – 2.08) |
| Age | 1.00 (0.97 – 1.04) |
| Modified Rankin Scale – 2-5 at 90 days | 0.95 (0.41 – 2.17) |
| Drug - tenecteplase | 0.98 (0.47 – 2.04) |
| Type of Enrolling Center – Comprehensive Stroke Center (CSC) | 1.83 (0.67 – 4.98) |
| Source - QUICR | 1.29 (0.61 – 2.73) |
| Large Vessel Occlusion on Computed Tomography Angiography (CTA) – Yes | 0.74 (0.22 – 2.47) |
| Onset to Needle Time | 1.00 (0.99 – 1.01) |
| Door to Needle Time | 1.00 (0.99 – 1.00) |
| EQ – Visual Analogue Scale (VAS) | 1.02 (1.00 – 1.04) |
| Length of Hospital Stay | 1.05 (0.97 – 1.13) |
| Baseline National Institutes of Health Stroke Scale (NIHSS) | 0.97 (0.90 – 1.04) |
| Computer Proficiency Score (CPQ) | **1.12 (1.04 – 1.21) *** |
| **Occlusion site on CTA (compared to patients with no visual occlusion on CTA)** |  |
| Left | 2.66 (0.82 – 8.63) |
| Right | 1.72 (0.57 – 5.15) |
| Bilateral | 1.03 (0.07 – 14.88) |
| Midline (Brainstem) | 1.20 (0.08 – 17.49) |
| **Ethnicity (compared to White)** |  |
| Ethnicity - Asian | 0.61 (0.17 – 2.19) |
| Ethnicity – Other Visible Minority | 0.52 (0.17 – 1.63) |
| **Education (compared to patients with no certificate, diploma or degree)** |  |
| Education – Graduate degree and higher | 0.43 (0.06 – 2.95) |
| Education – Undergraduate degree or college diploma | 0.49 (0.10 – 2.40) |
| Education – Some university/college, no diploma or degree | 0.37 (0.07 – 1.88) |
| Education – High school diploma or equivalent | 0.27 (0.05 – 1.29) |

***Significant differences (p < 0.05) are bolded**

**Section 2: Structured Qualitative Interview and Exit Survey**

**Methods**

**a.Structured brief qualitative interview to characterize preferences on computer cognitive assessment**

After completing Creyos, either in full (A) or partially (B), brief structured qualitative interviews were conducted with assessors (SS or PP). These questions were designed to quickly gather concise information about factors that facilitated assessment completion or reasons for discontinuation from the computerized cognitive assessment. Both interview sets contained the same questions, except for the second question, which enabled comparison between the groups. The structure interviews aimed to minimize patient burden and burnout by avoiding lengthy open-ended discussions.

The interviews were reviewed by two assessors (SS & PP) to first identify subthemes (child codes) – defined as recurring ideas or topics frequently mentioned in participant responses. Subsequently, subthemes were grouped into broader category themes (parent codes) through collaboration with a qualitative scientist (KND). This analysis employed an inductive approach, deriving concepts and themes directly from the raw interview data. Additionally, content analysis was used to quantify the frequency with which parent codes were identified in interviews.

A third group (C), included participants who never started the Creyos assessment. For these participants, a brief exit survey was administered. Since they had already declined more detailed assessments, we, and our institutional research ethics board, felt that a single, multiple-select question was preferable to a longer exit interview in this group, to avoid placing additional burden on the participants who had declined additional computer testing. The exit survey aimed to identify the most appropriate reason(s) for declining computerized testing. The variable list was informed by previous literature on participation in clinical trails and barriers to computerized testing.^1–5^ Ultimately, the goal was to identify and characterize common barriers to use of online cognitive assessments as a secondary endpoint in stroke RCT’s. Survey responses were tallied and visually represented through graphs.

A code book (Supplementary Table 4) was developed using thematic analysis of the brief structured qualitative interview from groups a) and b). Independently both assessors (SS & PP) reviewed the interview responses and identified sections that reflected key concepts related to cognitive testing and patient preference that were mentioned more than once in interviews.

**b. Structured Qualitative Interview (A and B) and Exit Survey (C) Questions**

1. **Those that successfully complete all assessments:**
2. What did you think about the online method of testing?
3. *Were you able to complete this by yourself or did someone have to help you get to the tests*?
4. What did you think about the online method of testing?
5. Were there any difficulties in carrying out the tasks?
6. Have you ever done in-person cognitive testing? If so do you prefer this online method or in-person?
7. How did you find the video and practise tutorial sessions prior to starting each task?
8. Any other feedback you would like to share with us today?
9. **Those that began the online tasks but did not finish all assessments:**
10. What did you think about the online method of testing?
11. *Was there a particular reason for not completing the online assessments?*
12. What did you think about the online method of testing?
13. Were there any difficulties in carrying out the tasks?
14. Have you ever done in-person cognitive testing? If so do you prefer this online method or in-person?
15. How did you find the video and practise tutorial sessions prior to starting each task?
16. Any other feedback you would like to share with us today?
17. **Those that decline online cognitive testing (brief exit interview with one multiple-select question:**

**1. We’d like to better understand the things that make it hard for people to use these tasks. Is ther any particular reason that you decided not to complete the online task?** Participants are asked **to choose as many options** that relate to them.

1. Uncomfortable with computers
2. Time demanding
3. No therapeutic benefits
4. Not interested
5. Lack of assistance
6. Research fatigue
7. Lack of incentives
8. Other

**Results**

**Brief Structured Qualitative Interview and Exit Survey Results**

A total of 242 people completed Creyos, and 76 were interviewed (group A). 7 people started but did not complete Creyos, and 3 were interviewed (group B). Of those that did not complete Creyos at all, 43 declined but responded to the exit survey (group C), and 37 agreed to complete the online cognitive assessment but were lost to follow-up (main paper, Figure 1).

Through this process, the following subthemes (child codes) were mentioned more than once in participant interviews: improved accessibility, improved comfort, inclusion of visual aids, dislike for computers, preference for social interaction, need for continuous feedback, limitation in understanding, motivational deficits, perceived difficulty of the task, physical limitation, technical difficulties, environment distractions and the need for assistance. Subthemes with similar experiential patterns were grouped together to form overarching parent codes (Supplemental Table 4), which included: reasons for preferring online cognitive testing, reasons for preferring in person cognitive testing and barriers to performance.

Content analysis on the “barriers to performance” parent code (Supplemental Figure 1) in patients who had completed the computer cognitive testing revealed that 45% of the structured interviews reported a perceived difficulty in tasks as a significant barrier. Other barriers were reported at rates less than 5%.

The exit surveys completed by participants who declined to complete the online cognitive battery (Group C) revealed barriers to computerized cognitive follow-ups (Supplemental Figure 2). The most frequently identified barriers included discomfort with computers (49%), lack of interest in completing non-therapeutic assessments (47%), and perceived time-demanding nature of the follow-up (44%).

**Supplemental Table 3: Qualitative Interview Patient Demographics**

|  | **Declined (**n=43) | **Completed** (n=76) | **Started but did not complete**  (n=3) |
| --- | --- | --- | --- |
| Age (mean (SD)) | **72 (11)** | **66.3 (13)** | **76.7 (6.8)** |
| Sex (% Female) | 20 (47%) | 34 (45%) | 1 (33%) |
| Drug Allocation (% tenecteplase) | 22 (51%) | 42 (55%) | 2 (67%) |
| Modified Rankin Scale (% 2-5) | 21 (49%) | 35 (46%) | 2 (67%) |
| Time from onset-to-needle, mean (SD), mins | 141 (64) | 138 (63) | 150 (34) |
| Time from door-to-needle, mean (SD), mins | 41 (23) | 39 (20) | 36 (13) |
| EQ-5D- Visual Analogue Scale (VAS) (mean (SD)) | 71 (22) | 74 (17) | 60 (27) |
| Length of Hospital Stay (mean (SD)) | **11 (9.7)** | **6.7 (6.6)** | **5.3 (3.5)** |
| Pre-treatment National Institutes of Health Stroke Scale (NIHSS) (mean (SD)) | 9.5 (5.8) | 10 (6.3) | 9.7 (4.5) |
| Type of Stroke center (% Comprehensive center) | 35 (81%) | 70 (92%) | 2 (67%) |
| Registry (% QUICR) | 31 (72%) | 47 (62%) | 3 (100%) |
| Large Vessel Occlusion on Computed Tomography Angiography (CTA) (% Yes) | 17 (40%) | 32 (42%) | 1 (33%) |
| Occlusion Side on CTA (%) | | | |
| No occlusion seen on CTA | 11 (26%) | 29 (38%) | 1 (33%) |
| Left | 13 (30%) | 19 (25%) | 1 (33%) |
| Right | 17 (40%) | 26 (34%) | 1 (33%) |
| Bilateral | 0 (0%) | 0 (0%) | 0 (0%) |
| Midbrain | 1 (2.3%) | 2 (2.6%) | 0 (0%) |
| Missing | 1 (2.3%) | 0 (0%) | 0 (0%) |
| Occlusion Location on CTA (%) |  |  |  |
| No occlusion seen on CTA | 14 (33%) | 38 (50%) | 1 (33%) |
| Distal occlusion | 10 (23%) | 9 (12%) | 0 (0.0%) |
| M2 Middle Cerebral Artery (MCA) occlusion | 9 (21%) | 9 (12%) | 1 (33%) |
| M1 MCA occlusion | 10 (23%) | 20 (26%) | 1 (33%) |
| Ethnicity (%) |  |  |  |
| White | 34 (79%) | 63 (83%) | 2 (67%) |
| Asian | 3 (7.0%) | 5 (6.6%) | 0 (0%) |
| Other Visible Minority | 6 (14%) | 8 (11%) | 1 (3.3%) |
| Education (%) |  |  |  |
| Graduate degree or higher | 2 (4.7%) | 6 (7.9%) | 0 (0%) |
| Undergraduate degree/college diploma | 13 (30%) | 29 (38%) | 1 (33%) |
| Some university/college, no diploma or degree | 5 (11%) | 8 (10%) | 1 (33%) |
| High school diploma or equivalent | 9 (21%) | 23 (30%) | 0 (0%) |
| No certificate, diploma or degree | 13 (30%) | 10 (13%) | 0 (0%) |
| Missing | 1 (2.3%) | 0 (0%) | 1 (33%) |

***Significant differences (p < 0.05) are bolded**

**Supplemental Table 4:** Brief structured interview codebook developed using a thematic analysis.

| **Parent Code** | **Child Code** | **Description** |
| --- | --- | --- |
| Reasons for preferring online cognitive testing | Improved Accessibility  *“For these types of testing, I prefer to do it online, as it gives me the flexibility to do it anytime”* (Male, 88 years old)  *“I prefer online method of testing as it is more accessible”* (Male, 69 years old) | Participants who preferred not having to travel to the hospital, pay for travel expenses and spend time waiting to see the physician but could instead perform the task when it best fit their schedule. |
|  | Improved Comfort  *“I’m not much of a conversationalist and preferred to complete the testing at home in comfort”* (Female, 84 years old)  *“The in-person tests are very stressful for me, and I prefer to do it at home, at my own comfort”* (Female, 66 years old) | Participants who felt more comfortable completing the task at home, in a familiar environment without the pressure of someone watching. |
|  | Inclusion of visual aids  *“Practice sessions were helpful in preparing for the actual test”* (Female , 71 years old)  *“I enjoyed that it wasn’t just written instructions but with visual aids which made it easier to understand”* (Female, 49 years old) | Participants who reported that the inclusion of a tutorial with visual aids and example questions with feedback improved their understanding of the task. |
| Reasons for preferring in person cognitive testing | Dislike for computers  *“I just don’t enjoy working on computers, if this was done in-person, I would be more than happy to finish the tests”* (Male, 82 years old) | Participants who have difficulties with technology. |
|  | Like social interaction  *“I personally don’t mind doing online testing, but I really enjoy doing in-person testing with real interaction”* (Female, 88 years old) | Participants who like the social interaction with research staff that comes with in-person research. |
|  | Continuous feedback  *“I enjoy talking to people in-person, and would appreciate reassurance and feedback after the test”* (Female, 82 years old)  *“I need feedback and in-person testing would be able to guide me and provide assistance if required”* (Male, 69 years old) | Participants who reported that the presence of a staff member to ask questions of would have improved their performance. |
| Barriers to performance | Limitations in Understanding    *“The questions were very hard to understand, and most of them were confusing, and the practice was not that helpful”* (Male, 83 years old)  *“If I took my time to understand the task, I would have done better, and not find it difficult”* (Male, 78 years old)  *“I found the test frustrating, as some of the test were really difficulty to understand”* (Male, 77 years old) | Participants who did not understand the instructions, or did not find the tutorials helpful, and felt this limited their performance. |
|  | Motivational Deficits  *“I was able to do most of the test by myself but had lost hope and stopped after I was getting frustrated with tasks with a time limits”* (Male, 79 years old)  *“I rushed through it quickly, didn’t really follow instructions… wasn’t in the right mindset”* (Male, 71 years old) | Participants who felt as though they had no reason to put their best efforts into the task and thus performed poorly. |
|  | Perceived Difficulty of the task  *“One of the tasks was very confusing and I was impatient and rushed through most of the questions”* (Male, 69 years old) | Participants who perceived the task as beyond their cognitive abilities and thus did not complete or put their best effort into the task. |
|  | Physical Limitations  *“I didn’t find the questions hard, but I have a hard time using the computer mouse, so it frustrated me as I couldn’t respond quickly”* (Male, 70 years old) | Participants who were unable to complete or properly perform the computer tasks due to medical or physical limitations. |
|  | Technical Difficulties  *“Had some technical difficulties as I use an older computer with an older version of Firefox, but was able to try again on another computer”* (Male, 77 years old) | Participants who were not able to access the testing site, had software incompatibilities or did not have reliable access to internet or WiFi. |
|  | Environmental Distractions  *“I heard some background noises while doing the online test, so it threw me off a bit, but was able to complete the whole test”* (Female, 49 years old) | Participants who reported that the presence of external sounds, visual stimuli or other persons disrupted their performance on the task. |
|  | Required Assistance  *“At my current age, online testing isn’t my forte, and I needed help setting up and completing the test”* (Male, 82 years old)  *“My wife was with me the whole time, in case I needed any help with the questions”* (Male, 73 years old)  *“It would be really helpful to have someone beside me to explain some of the rules”* (Male, 71 years old) | Participants were not able to independently access and complete the task and required assistance from another individual due to physical, cognitive or technological limitations |

5%

4%

4%

1%

3%

45%

**Supplemental Figure 1:** Frequency distribution of participants self-reported barriers to performance (n=76)

0%

0%

49%

9%

19%

14%

47%

44%

**Supplemental Figure 2:** Frequency distribution of the participants self-reported reason for declining participation (n=43)

**References**

1. Peel R, Ren S, Hure A, et al. Evaluating recruitment strategies for AUSPICE, a large Australian community-based randomised controlled trial. *Medical Journal of Australia*. 2019;210(9):409-415. doi:10.5694/mja2.50117

2. Kaur G, Smyth RL, Powell CVE, Williamson P. A survey of facilitators and barriers to recruitment to the magnetic trial. *Trials*. 2016;17(1):1-10. doi:10.1186/s13063-016-1724-3

3. Newberry A, Sherwood P, Hricik A, et al. Understanding recruitment and retention in neurological research. *Journal of Neuroscience Nursing*. 2010;42(1):47-57. doi:10.1097/JNN.0b013e3181c1fdd9

4. Newington L, Metcalfe A. Factors influencing recruitment to research: Qualitative study of the experiences and perceptions of research teams. *BMC Med Res Methodol*. 2014;14(1). doi:10.1186/1471-2288-14-10

5. Zhang Q, Wei JH, Fu X, et al. Can we trust computers to assess the cognition of stroke patients? A systematic review. *Front Neurol*. 2023;14:1180664. doi:10.3389/FNEUR.2023.1180664/FULL
